# With thanks to our 2023 peer reviewers

**DOI:** 10.24095/hpcdp.44.3.07

**Published:** 2024-03

We are grateful to the following individuals for their significant contribution to *Health Promotion and Chronic Disease Prevention in Canada* as peer reviewers in 2023. Their expertise ensures the quality of our journal and promotes the sharing of new knowledge among peers in Canada and internationally.

Katrine Aadland

Sarah Abdunnabi

Isabel Aguilar-Palacio

Josphine Aho

Samia Amin

Sharon Anderson

Michael Armstrong

Rachelle Ashcroft

Nathalie Auger

Thilina Bandara

Kate Battista

Maulik Baxi

Amy Beardmore

Emilie Beaulieu

Nicole Blackman

Songul Bozat-Emre

Lauren Bresee

Andrea Brown

Mark A. Canfield

Michael Cantinotti

Jude Mary Cnat

Allison Crawford

Lise Dassieu

Justin T. Denney

Sarah Dermody

Chris Drinkwater

Todd Duhamel

Rachel Engler-Stringer

Jillian Evans

Andre-Anne Fafard St-Germain

Robert Flynn

Natalia Formento

Natalie Frandsen

Sarah Fraser

Allison Glasser

Jacey Greece

Paul Hbert

Gerri Hirschfeld

Winston Husbands

Tay Jeong

Jodi Kalubi

Yvonne Kelly

Babak Khoshnood

Sara Kirk

Laurence Kirmayer

Sarah Larney

Kalan Leaks

Lisa M. Lix

Jinjin Lu

Yona Lunsky

Martha L. MacLeod

Jakob Manthey

Krystle Martin

Spenser Martin

Katerina Maximova

Ted McDonald

Ellen McGarity-Shipley

Nicole Milano

Ethan Morgan

Howard Morrison

Caitlin Muhl

Patti-Jean Naylor

Assumpta Ndengeyingoma

Kwok Ng

Yeshambel Nigatu

Chelsea Noel

MaryAnn Notarianni

Dominik Nowak

Melissa O’Donnell

Timothy Olds

Roxy O’Rourke

Heather Orpana

Candice Oster

Karen Patte

Scott Patten

Kaitlyn Patterson

Anne Perozziello

Shameka Phillips

Daria Piacentino

Florence Pich

William Pickett

Emmanuelle Pirard

Nathaniel Pollock

Nashira Popovic

Shirley Quach

Walter Roberts

Laura Rosella

Michelle Ruhigisha

Zainab Samaan

Zouina Sarfraz

Travis Saunders

Deborah Scharf

Robert Schlack

Ruth Schofield

Leah Sharman

Jay Shaw

Suzanne Sicchia

Isabelle Simard

Manish M. Sood

Edward G. Spilg

Clifford Stevenson

Stephanie Toigo

Mark Tremblay

Alexandra Uhrig

Nicole Valentine

David E. Vance

Lee Verweell

Georgina Warner

Colleen Webber

Robert Wellman

Erica Wennberg

Colin Wilbur

Christina Wolfson

Suzy Wong

Scott Yamamoto

Renate Ysseldyk

He Zhu

Jillian Zitars

Jennifer D. Zwicker

